# Recovery from rat sciatic nerve injury *in vivo* through the use of differentiated MDSCs *in vitro*

**DOI:** 10.3892/etm.2012.785

**Published:** 2012-10-31

**Authors:** XIANGYI ZENG, LI ZHANG, LIANG SUN, DAI ZHANG, HENGWU ZHAO, JUN JIA, WEI WANG

**Affiliations:** 1 The Third Affiliated Hospital of Liaoning Medical University;; 2 Jinzhou Clinical Institute of Liaoning Medical University/Jinzhou Central Hospital;; 3 Liaoning Medical University, Jinzhou, Liaoning 121000, P.R. China

**Keywords:** muscle-derived stem cell, epineurium, sciatic nerve function index, recovery rate of gastrocnemius muscle wet weight

## Abstract

In this study, muscle-derived stem cells (MDSCs) whose differentiation into neuron-like cells was induced by ciliary neurotrophic factor (CNTF) and *Salvia (Salvia miltiorrhiza) in vitro* were used to repair rat sciatic nerve injuries *in vivo,* in order to investigate their multifunctional characteristics as pluripotent stem cells. The sciatic nerve in the right side of the lower limb was exposed under the anesthetized condition of 10% chloral hydrate (0.3 ml/100 g) injection into the abdominal cavity. The tissue which was 0.5 cm above the sciatic nerve bifurcation was broken using a hemostat. After induction, MDSCs were transferred in sodium hyaluronate gel and were placed into the damaged area. An untreated control group was also included in this study. The surgical area was sutured after washing with gentamycin sulfate solution. Sciatic nerve function index (SFI) was calculated, electrophysiological tests were performed and the recovery rate of gastrocnemius muscle wet weight was also calculated. Four weeks post-surgery, the SFI and the recovery rate of gastrocnemius muscle wet weight in the MDSC group were significantly higher than those in the control group (P<0.05). MDSCs whose differentiation is induced by CNTF and *Salvia* play an active role in the repair of peripheral nerve injury.

## Introduction

Limb joint and soft tissue injuries are among the most common conditions in clinical practice and are often followed by peripheral nerve injury in 5% of cases. The peripheral nerve is a mixed nerve, and nerve fibers emanating from one side cross over to the other within nerve bundles. Therefore, the complete recovery rate is only 10–25% after injury and treatment of peripheral nerve injuries remains a challenge worldwide.

Several researchers have performed numerous studies in this field and proposed that the basic condition for peripheral nerve repair is the construction of the nerve regenerative chamber, which can create a specific microenvironment for regeneration of the nerve (1,2). As stem cell research has developed, study into novel tissue engineering seed cells has received increasing attention. Muscle-derived stem cells (MDSCs) originate from muscle tissue. In addition to aspects shared by stem cells, MDSCs also exhibit such characteristics as harmlessness to the body, a wide range of sources, ready acceptance by patients, lack of restrictions of source of donor, lack of immunosuppressive effects and fewer ethical restrictions. MDSCs are therefore ideal seed cells for peripheral nerve repair (3–6). As the study of Chinese traditional medicine has developed, it has been confirmed that *Salvia* (*Salvia miltiorrhiza*) may be used to induce the differentiation of multi-functional stem cells into neuron-like cells (7–10).

In the present study, MDSCs whose differentiation was induced by ciliary neurotrophic factor (CNTF) and *Salvia* were used to repair sciatic nerve injury in rats in order to further confirm their potential as pluripotent stem cells. This study may contribute to a theoretical concept of more latent cytokines and novel seed cells for the construction of tissue engineered peripheral nerve grafts.

## Materials and methods

### 

#### Experimental animals

Adult Sprague-Dawley (SD) rats (n=12) were divided into 2 groups. The sciatic nerve in the right lower limb was exposed under the anesthetized condition of 10% chloral hydrate (0.3 ml/100 g) injection into the abdominal cavity. The tissue, which was 0.5 cm above the sciatic nerve bifurcation, was broken using a hemostat. After induction, MDSCs were transferred to sodium hyaluronate gel and placed into the damaged area. An untreated control group was also included in this study. The surgical area was sutured after washing with gentamycin sulfate solution. Animal experiments were performed in accordance with the Guide for the Care and Use of Laboratory Animals.

### Experimental materials

#### General observation

The recovery of the wound and the formation of ulcers in the plantar region were recorded. Under mild anesthesia, the sensory function recovery was examined following plantar puncture.

#### Sciatic nerve function index (SFI)

Four weeks after surgery, SFI was calculated using the method described by Reynolds and Weiss (11). Hind legs of the rats were dyed with ink. When the rats walked on the surface of one piece of white paper, the footprints of healthy feet (N) and wounded feet (E) were measured in 3 indices as follows: length of footprint (IPL, from toe to heel), width of toes (ITS, from the 1st to the 5th toe) and width of middle toes (IIT, from the 2nd to the 4th toe). The results should be accurate to 0.1 mm. SFI was calculated according to the formula described by Bain *et al*(12): SFI = −38.3 [(EPL - NPL)/NPL] + 109.5 [(ETS - NTS)/NTS] + 13.3 [(EIT - NIT)/NIT] −8.8. A SFI value between 0 and 11% represented normal nerve function, whereas −100% represented complete damage of nerve function, and −11 to −100% incomplete nerve function recovery.

#### Electrophysiology test

Four weeks after surgery, the sciatic nerve at the surgical site was exposed under anesthesia. The transplanted section was linked with an electrode and the gastrocnemius was linked with a recording electrode to determine the action potential (AP) and mean conductive velocity (MCV) of the sciatic nerve.

#### Recovery rate of gastrocnemius wet weight

Four weeks following surgery, the gastrocnemius was completely removed and its wet weight was then measured by ER-182 electronic balance (1/2,000 g) to calculate its recovery rate.

#### Statistical analysis

All statistic data are presented as the means ± SD. statistical analyses were performed using SPSS 10.0 software. A P-value <0.05 was considered to indicate a significant difference and P<0.01 a statistically significant difference.

## Results

### 

#### General observation

Following surgery it was found that the toes of the rats were crooked and muscle contraction was not observed when the toes were punctured. One week after surgery, it was found that the surgical area had recovered well without infection. However, there was redness and swelling on the skin of the right foot and weight-bearing area. All the rats were inactive and the operated legs were immobile. Two weeks after surgery, ulcers were observed on the skin behind the ankle of the operated area in some rats, while other rats exhibited redness and swelling on the soles of their feet. There were no obvious differences between the MDSC and control groups.

Three weeks after surgery, the rats’ moods had recovered and the treated legs became active. However, the toes and knee joint remained crooked with various degrees of muscle shrinkage in the treated legs. At the same time, inflammation and ulcers were found in the plantar region. Four weeks after surgery, the knee joints of all the rats remained crooked; however, the inflamed area and ulcers in the plantar region were no longer observed. All the rats reacted when their toes were punctured. In the MDSC group, there were 3 rats that generally recovered from inflammation and ulcers, and partly recovered from muscle shrinkage. However, all the rats in the control group exhibited various degrees of muscle shrinkage in the treated legs.

#### SFI

**Figure 1:**
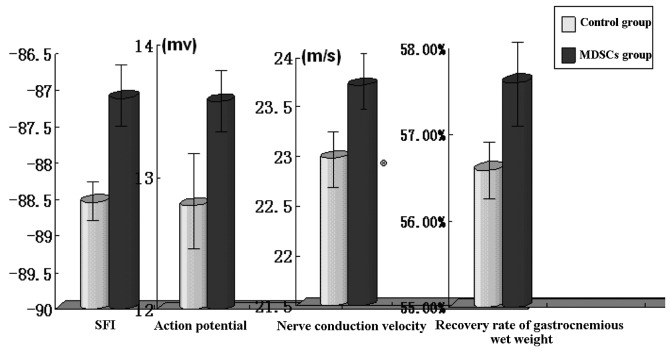
Sciatic nerve function index (SFI), action potential, nerve conductive velocity and recovery rate of gastrocnemius wet weight in the MDSC and control groups.

SFI was statistically higher in the MDSC group in comparison with the control group (P<0.05; Table I and Fig. 1).

#### Electrophysiology test

Sciatic action potential and nerve conductive velocity were statistically higher in the MDSC group in comparison with the control group (P<0.05; Table I and Fig. 1).

#### Recovery rate of gastrocnemius wet weight

The recovery rate of gastrocnemius wet weight was statistically higher in the MDSC group in comparison with the control group (P<0.05; Table I and Fig. 1).

## Discussion

In Chinese traditional medicine, peripheral nerve injury is considered to belong to the category of flaccidity. Flaccidity is a syndrome with such symptoms as weak tendons and feeble muscle, leading to muscle shrinkage due to lack of bodily exercise.

Nerve damage can cause blood stasis and meridian obstruction, leading to poor circulation and malnutrition. Bodily functions therefore exhibit anomalous changes and pathological changes occur in such a situation.

Modern experimental research has confirmed that medicine promoting blood circulation to remove blood stasis can achieve favorable results in the treatment of disease caused by peripheral nerve injury. Qian *et al*(13) demonstrated that Bu Yang Huan Wu Tang reduces the shrinkage of neurons in injured peripheral nerve and aids nerve recovery. Fang *et al*(14) confirmed that such compound Chinese herbal medicine as ginseng, *Astragalus* and *Salvia* can promote peripheral nerve regeneration.

As a result of technical developments in genetic engineering, cells with biological activity are used as carriers for nutritional nerve factor. After modification, the carrier cells can constantly provide nutritional nerve factor to the gene, which provides a prospective application for nerve restoration and regeneration with nutritional nerve factor and which also gives hope to nerve tissue engineering of cell types.

MDSCs originate from muscle. They are precursor cells of skeletal muscle cells. Compared with other cells, they exhibit such characteristics as partial differentiation ability, favorable histocompatibility, harmlessness to the body, wide range of sources and ready acceptance by patients. Several investigators have noted that MDSCs originating from skeletal muscle have the characteristics of stem cells whose differentiation is induced by CNTF (15–26). This study used the liquid culture method allowing the generation of a large number of MDSCs and the induction of MDSCs to obtain amplification ability. MDSCs after the 3rd generation may be used for cell differentiation and cell treatment. As seed cells, MDSCs play a substantial role in the restoration of rat sciatic nerve injury and regeneration of the peripheral nerve under the effect of *Salvia*.

Three elements of nerve tissue engineering include seed cells, nerve carrier and nerve nutritional factor. The source and large proliferation of seed cells are the most important problems to be solved. In addition, other research on bionic scaffold material has received increasing attention. Research on nerve carriers has also made substantial progress.

Hyaluronic acid is a linear polysaccharide macromolecular material, which comprises (1→4)-D-glucuronic acid-β (1→3)-N-acetyl glucosamine disaccharide units and is widely present in the connective tissue of humans and animals. With a 3-dimensional network structure formed by interaction between molecules, it has such characteristics as maintaining tissue formation, important psychological function and good viscoelasticity. Glucuronic acid is also a main element in compounding cellular and extracellular matrices, which occupy vital roles in maintaining normal physiological function, supporting the growth and differentiation of cells and restoration of injured tissue. In the early period of wounding, such cells as fibroblast cells and macrophages exhibit a marked increase in compounding glucuronic acid in the function of growth factor. It has been shown in the comparison between the embryo before and after birth that glucuronic acid maintains high thickness, which prevents collagenous fibrous tissue from contracting and even aids tissue recovery without any cicatricial sign. After birth, glucuronic acid remains at high thickness in the early period; later it is gradually dissolved by hyaluronidase. At the same time, the combination of collagenous fiber increases. Finally, cicatricial restoration is formed. Additionally, glucuronic acid has such functions as promoting blood vessel growth, partly improving circulation of injured areas and aiding growth factor expression. There are high quantities of carboxyl and hydroxyl in glucuronic acid molecules. These two elements can form an intermolecular and intramolecular hydrogen bond in water with such potency that it can hold 1,000 times its weight in water (27,28).

In the present study, sodium hyaluronate gel was used as a cell carrier. In the recovery process, the extracellular matrix had an effect on cell performance. Cells also modified the extracellular matrix in various ways, forming the micro-environment. The whole recovery process, which was constantly changing, supported the growth of the cells. In conclusion, the restoration and regeneration of tissue is a process in which cells composed of different molecules and macromolecules proliferate, differentiate and reconstruct the matrix.

## Figures and Tables

**Table I:** Experimental data in the MDSC and control group.

Group	SFI	Action potential	Nerve conductive velocity	Recovery rate of gastrocnemius muscle wet weight (%)
MDSC	−87.11±0.88^a^	13.58±0.53^a^	23.72±0.62^a^	57.6±0.0094^a^
Control	−88.54±0.64	12.79±0.69	22.99±0.61	56.59±0.0076

a P<0.05. SFI, sciatic nerve function index.
